# Machine learning approaches in Covid-19 severity risk prediction in Morocco

**DOI:** 10.1186/s40537-021-00557-0

**Published:** 2022-01-06

**Authors:** Mariam Laatifi, Samira Douzi, Abdelaziz Bouklouz, Hind Ezzine, Jaafar Jaafari, Younes Zaid, Bouabid El Ouahidi, Mariam Naciri

**Affiliations:** 1 Department of Biology, Faculty of Sciences, Mohammed V University, Rabat, Morocco; 2 FMPR, University Mohammed V, Rabat, Morocco; 3 Laboratory of Pharmacology and Toxicology, Faculty of Medicine and Pharmacy, Rabat, Morocco; 4 FSTM, University Hassan II, Casablanca, Morocco; 5 Research Center of Abulcasis University of Health Sciences, Cheikh Zaïd Hospital, Rabat, Morocco; 6 Department of Computer Science, Faculty of Sciences, Mohammed V University, Rabat, Morocco

**Keywords:** COVID-19, Severity, Machine learning, Feature selection, Feature reduction, Data analysis

## Abstract

The purpose of this study is to develop and test machine learning-based models for COVID-19 severity prediction. COVID-19 test samples from 337 COVID-19 positive patients at Cheikh Zaid Hospital were grouped according to the severity of their illness. Ours is the first study to estimate illness severity by combining biological and non-biological data from patients with COVID-19. Moreover the use of ML for therapeutic purposes in Morocco is currently restricted, and ours is the first study to investigate the severity of COVID-19. When data analysis approaches were used to uncover patterns and essential characteristics in the data, C-reactive protein, platelets, and D-dimers were determined to be the most associated to COVID-19 severity prediction. In this research, many data reduction algorithms were used, and Machine Learning models were trained to predict the severity of sickness using patient data. A new feature engineering method based on topological data analysis called Uniform Manifold Approximation and Projection (UMAP) shown that it achieves better results. It has 100% accuracy, specificity, sensitivity, and ROC curve in conducting a prognostic prediction using different machine learning classifiers such as X_GBoost, AdaBoost, Random Forest, and ExtraTrees. The proposed approach aims to assist hospitals and medical facilities in determining who should be seen first and who has a higher priority for admission to the hospital.

## Introduction

The World Health Organization (WHO) proclaimed the Coronavirus COVID-19 a public health emergency with pandemic potential on March 11, 2020 [1]. The pandemic’s rapid spread has caused chaos and necessitated quick responses to mitigate the damage. All positive COVID-19 cases have been required to be hospitalized from the beginning of the pandemic, regardless of the severity of the sickness and with the significant increase in cases worldwide, hospitals have reached 100% occupancy, causing medical facilities to be overburdened [2]. Thus, having techniques allowing rapid identification of patients at high risk of severe and non-severe forms for prioritization hospitalization is critical [3].

The SARS-CoV-2 virus RNA test is currently used to diagnose COVID-19 [4]. This is a qualitative test that evaluates whether the patient is infected with the virus. CT scans are a useful tool for diagnosing COVID-19. However, roughly 20% of COVID-19 patients had no evident imaging alterations in their lungs [5]. Furthermore, CT presentsa number of drawbacks, including unnecessary irradiation and the misuse of a limited resource for the purpose of screening [6]. Despite the availability of protein-based antibody and antigen tests with quicker turnaround times, there are still concerns about their accuracy [7].

Common laboratory procedures, such as total blood cell count, blood biochemistry, and immunological testing, offer a viable alternative to SARS-CoV-2 diagnosis. In fact, several investigations have found that COVID-19 patients had reduced white blood cell, lymphocyte, and platelet counts [5, 8] as well as high serum ferritin and C-reactive protein (CRP) levels [9]. According to Wynants et al. [7] several clinical characteristics, including age, gender, lactic dehydrogenase (LDH), C-reactive protein (CRP), and lymphocyte count, are significantly associated with the severity of COVID-19 individuals. Furthermore,a report released recently by a Chinese team discovered that three important indicators (LDH, CRP, and lymphocyte) can be used to predict COVID-19 mortality with over 90% of accuracy [10]. Thus,we hypothesize that using Machine Learning to classify severity and assess prognosis for COVID-19 patients across a variety of routinely performed laboratory tests may be advantageous. In fact, Machine Learning (ML) has been shown to be a useful technique for supporting caregivers in medical decision-making, and it has been utilized in multiple COVID-19 studies [11–13] to construct a model that compares positive and negative SARS-CoV-2 patients. Other research [14–17] has focused on COVID-19 detection, prediction, and treatment formulation [18, 19]. Moreover, in many cases, no additional material expenditure is necessary because the necessary information is already contained in the patients’ medical records. This would make it possible to examine a large number of patients in a short period of time. Findings may be generated in near real-time.

Several researchers have attempted to use machine learning to predict the severity of Covid 19. Pourhomayoun and Shakibi [2] employed a variety of machine learning techniques to predict the mortality risk of COVID-19 patients, including Support Vector Machine (SVM), Artificial Neural Networks, Random Forest, Decision Tree, Logistic Regression, and K-Nearest Neighbour (KNN). The Neural Network method had the best performances in predicting the mortality rate, with an overall accuracy of 89.98%. The goal of Vaishya et al. was to identify seven key AI applications for the COVID-19 pandemic. By collecting and evaluating all past data, they demonstrate that AI plays a vital role in detecting clusters of cases and predicting where this virus would affect in the future [20]. Zhou et al. use a machine learning model to predict the evolution of illness severity based on a cohort of training, validation, and internal test sets. In the feature selection step they use a genetic algorithm (GA) [21] and SVM algorithm forprediction. Wungu et al. used ML to investigate the link between different cardiac indicators and the severity/mortality of COVID-19 patients [22]; they conclude that High CK-MB, PCT, NT-proBNP, BNP, and d-dimer could be predictive markers for severity of COVID-19. Cai et al. wanted to see how CT measurement of COVID-19 pneumonia affected disease severity assessment and clinical outcome prediction in COVID-19 patients [23]. The severity of the disease was divided into three categories: moderate, severe, and critical. They created random forest (RF) models for classification and regression in order to determine the severity of the condition (Moderate, Severe, and Critical). In the classification of moderate vs. (severe + critical) and severe vs. critical, the AUCs of RF classifiers were 0.927% and 0.929%, respectively. The goal of Yaşar et al’s study [24] study was to use deep learning (DL), random forest (RF), and gradient boosted trees to categorize three COVID-19 positive patient groups (moderate, severe, and critical) and a control group based on blood protein profiling (GBTs). They found that RF had a greater accuracy rate (96.21%) than DL (94.73%). The ensemble classifier GBTs, on the other hand, generated the best results (96.98%). The two most important proteins linked with disease severity were TGB1BP2 in the cardiovascular II panel and MILR1 in the inflammatory panel. Banoei et al. [25] use statistical method SIMPLS to predict hospital mortalityand Latent class analysis (LCA) was carried to cluster the patients with COVID-19 to identify low- and high-risk patients.Using training and validation sets, the SIMPLS model was able to predict hospital mortality in patients with good accuracy (AUC > 0.85). Vafa Bayat et al. [26] use pairwise correlations to compress their dataset of 70 characteristics, and the X_GBoost model for prediction. They conclude that ferritin, CRP, LDH, and D-dimers may be used to detect SARS-CoV-2 infection. Yan et al. [27] established a model to predict COVID-19 patients’ criticality and mortality. The researchers used data from 375 patients (201 survivors) at Wuhan’s Tongji Hospital. The ML method X_GBoost was used, and it was found to be 93% accurate. LDH, lymphocytes, and high-sensitivity CRP were the important features for predicting mortality risk in this model. Wang et al. [28] used a data set of 296 patients from the First People’s Hospital of Jiangxia District in Wuhan, China, to predict severity in COVID-19 patients. The model was created using the ML technique X_GBoost. The clinical model had an AUC of 83% and was based on age, hypertension history, and coronary heart disease. Age, hs_CRP, oxygen saturation (SpO2), neutrophil and lymphocyte count, D-dimer, and AST were used to create the model. In the validation cohort, this model performed better, with an AUC of 88%. Hu et al. [29] built a machine learning algorithm for predicting COVID-19 patients’ mortality risk. The prediction model was built using data from 183 patients. According to the model’s performance, the researchers tried ten methods and chose five of them (LR, partial least squares (PLS) regression, elastic net (EN) model, RF, and bagged flexible discriminant analysis (FDA). According to the AUC, the LR model, RF, and bagged FDA all performed similarly. Because of its simplicity and interpretability, LR was chosen as the final model. The models used age, hs CRP level, lymphocyte count, and D-dimer level as the most important four variables. On the validation set, the model’s AUC, sensitivity, and specificity were 88.1%, 83.9%, and 79.4%, respectively.

**Fig. 1:**
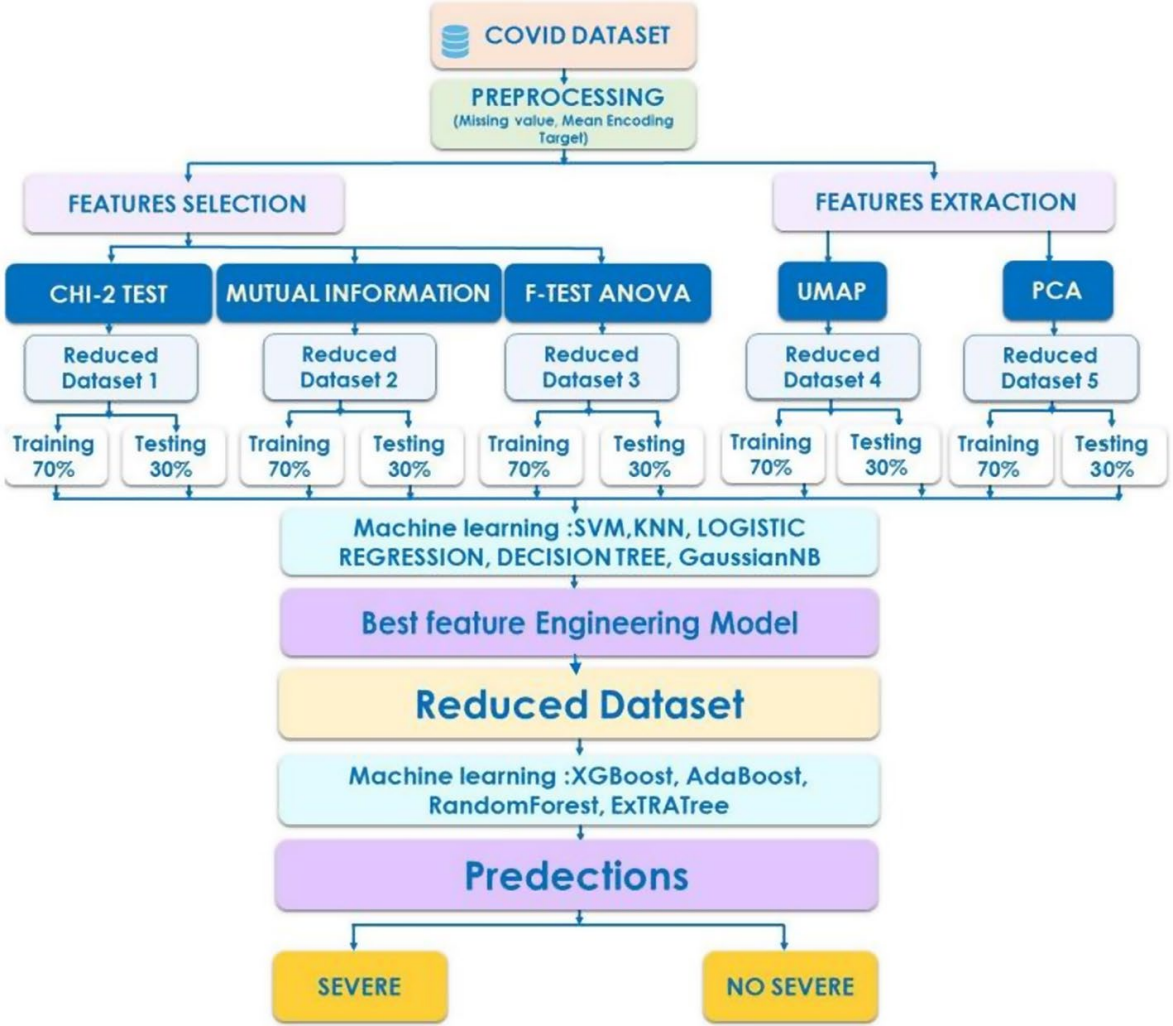
High-level system architecture

Using blood or urine test results, Yao et al. [30] established a model to predict the severity of COVID-19. The study included 137 patients from Huazhong University of Science and Technology’s Tongji Hospital (75 of them were critically ill). The severeness detection model was built using the machine learning technique SVM, which had an accuracy of 81.48%. Age, blood test values (neutrophil percentage, calcium, and monocyte percentage), and urine test values (urine protein, red blood cells (occult), and pH (urine) were the highest-ranking features found by the model.In patients with moderate COVID-19, Zhao et al. [31] created a model for predicting severity. LR models, both univariate and multivariate, were used to pick six important features from a total of 22. The prediction model was created using the SVM technique, and it had 0.91% accuracy, 0.90 sensitivity, and 0.94 specificity. IL-6, high-sensitivity cardiac troponin I (cTnI), procalcitonin, hs_CRP, chest discomfort, and calcium were the top six indicators for predicting severity (Fig. 1).

These studies have a number of drawbacks. First, due to the small sample size, some relevant signs and symptoms such as comorbidities and age were not found to be significant predictors of Covid 19 severity. Second, most studies do not use data feature engineering models, which is an important step in locating relevant features, and while some studies do use a reduction algorithm, we find that they are content to use only one method, which is typically a statistical method that produces poor predictions [19]. As a result, the data in this study was examined and tested utilizing five feature engineering models: Chi 2, Mutual information, F test Anova, PCA, and UMAP, a new feature extraction approach. These models were used to determine which features are most useful in predicting disease severity.

## Data description

### Patients’ samples

From July 1st to September 15th, 2020, 337 SARS-CoV-2 patients were hospitalized to the Cheikh Zaid Hospital for observational research. Nasopharyngeal swabs were taken on admission to the departments dedicated to managing of patients suspected of having COVID-19.The Coronavirus genomic material was detected at Cheikh Zaid laboratory, using real-time PCR, the reference method for the molecular diagnosis of SARS-CoV-2 [32, 33]. In addition, the patients had a chest CT examination at the time of admission. COVID-19 diagnosis was confirmed at the time of admission by reverse transcription polymerase chain reaction (RTPCR) analysis of samples from the nasopharyngeal swabs, and patients were subsequently divided into non-severe and severe COVID-19 groups on the basis of clinical criteria using the American Thoracic Society guidelines for community-acquired pneumonia. Briefly, severe COVID-19 patients showed significant lung damage and required mechanical oxygenation. The classification of the patients in our cohort into two groups (severe and non-severe). This allowed us to pinpoint the factors that would most likely serve as predictors of COVID-19 severity.

### Clinical laboratory examinations

Viral nucleic acid test by reverse transcription–polymerase chain reaction (RT-PCR) is the first line screening method of choice, biological and imaging markers that also contribute to the diagnosis of COVID-19, confirmation of this viral disease is done by identifying SARS-CoV-2 RNA in biological samples. The detection of the viral genome (RNA) in the upper airways (nasopharynx or oropharynx) is one of the mainstays of the diagnosis of SARS-CoV-2 infection and is done by analyzing the presence of the virus in a nasopharyngeal swab taken from a patient. In the laboratory, we use the “Berlin Protocol”, developed and made available worldwide in mid-January 2020 by Professor Christian Drosten, Director of the Institute of Virology at the Charite Hospital in Berlin. This test targets the E and RdRp gene of SARS-CoV-2. RT-qPCR is used to quantify the viral load in a sample and measure the evolution over time [34].

The hospital’s biology department conducted many analyses to acquire clinical data for this study. As a result, a database was created containing the variables listed in Table 1.

**Table 1:** Descriptive for the features considered in the present study

Variable name	Description
Severity classification	Severe or non-severe (Target: 194 severe and 146 non_severe)
Sex	Male or female
Age (years)	The patient age in years
Platelet	Elements found in the blood. They are best known for their role in blood clotting, and are activated in the event of vascular damage to stop bleeding
Lymphocyte	Elements found in the blood. They have an important role in the immunity process
PLR	They are considered prognostic factors in many inflammatory diseases, cardiovascular diseases and heart disease
ALT	Alanine aminotransferase is an enzyme necessary for the proper functioning of the body, allowing certain liver diseases to be identified or their progression to be monitored
AST	Aspartate aminotransferase is an enzyme normally found in the liver, heart and muscles. A high level of AST in the blood can be a sign of liver or heart damage, certain cancers or other diseases
LDH	Lactate dehydrogenase is an enzyme present in almost all tissues and organs of the human body: muscles, liver, lungs, red and white blood cells play an important role in the transformation of sugars into energy
D-dimers	They are the molecules resulting from the destruction of fibrin, a protein produced mainly during blood coagulation
C_reactive protein	It is a protein that appears in the blood during acute inflammation. Its level increases rapidly after the onset of inflammation
Weight	Patient weight
Comorbidities	Comorbidity refers to the combination of two diseases in one person, or the presence of one or more disorders that occur at the same time as a primary disease

### Ethic aspects

The Cheikh Zaid Foundation supported this study. Accordingly, it was approved by the Local Ethics Committee of Cheikh Zaid Hospital, Rabat, Morocco, Project: CEFCZ/PR/2020-PR04.

## Materials and methods

### Data processing: Mean Encoding Target

Mean Encoding Target is a method of substituting a category value with the target variable’s mean. From a mathematical approach, the Mean Encoding [35] represents the likelihood of the target variabledepending on each value of the feature. The transformation turns the value xi of a categorical property X to a scalar Siwhich represents a probability estimate for Y given X, and the encoded value wraps the target variable so that. The transformation’s formula is as follows:

$$\begin{aligned} x_{i} \rightarrow S_{i} = P(Y | X = x_{i}) \end{aligned}$$

$S_{i}$ reflects the modified attribute’s probability; it is automatically normalized between 0 and 1.

### Data visualization: RadViz visualizer

RadViz [36] is a multivariate data visualization algorithm that plots points on the interior of a circle, normalizing their values on the axes from the center to each arc, and then depicts each feature dimension equally around the circumference of a circle. This strategy is used to discover class separation, if there is a potential to learn from the feature set, or if there is just too much noise. It allows as many dimensions as can fit on a circle, greatly increasing the visualization’s dimensionality.

### Machine learning: feature engineering

Feature engineering is crucial since the number and quality of features in a dataset substantial impact on whether or not a model performs well in ML applications. Feature engineering is made up of two parts: feature selection and feature generation or extraction.

Feature extraction aims to develop more relevant features from the data’s current raw features in order to improve the learning algorithm’s predictive power, whereas feature selection is a critical problem in machine learning, where we will have multiple features in line and must choose the best features to build the model. We used ANOVA (Analysis of Variance), Chi-square test, and Principal component analysis (PCA) from the statistical field, Mutual Information from the theory information field, and UMAP from topological data analysis. These methods help us to solve the problem of feature selection by testing the relationship between the features and the response variables, and the best features are the features that are highly dependent on the response variable.

These feature engineering algorithms are utilized in this study to select the best set of features or components from all of the data. We’ll choose a feature engineering method that has a high performance rating from multiple ML Classifiers.

#### Mutual information

Mutual information determines how statistically dependent two variables are. It assigns a score to each characteristic based on how much information is communicated on average in one random variable about another. Thus, a high mutual information score between two variables suggests a significant reduction in uncertainty; a low mutual information score shows a minor reduction, and a zero mutual information score indicates that the variables are unrelated. Cover and Tomas [37] define the mutual information between two discrete variables X AND Y, abbreviated I(X; Y), as follows:

$$\begin{aligned} I(X;Y) = \sum _{x,y}^{}P_{XY}(x,y) log \frac{P_{XY}(x,y)}{P_{X}(x)P_{Y}(y)} = E_{P_{XY}} log \frac{P_{XY}}{P_{X}P_{Y}} \end{aligned}$$

Here ${P_{X}(x)}$ and ${P_{Y}(y)}$ are the marginals:

$$\begin{aligned} P_{X}(x) = \sum _{y}^{} P_{XY} (x) \end{aligned}$$

#### ANOVA F-statistic ensemble (AFSE)

ANOVA stands for “analysis of variance,” It is a parametric statistical hypothesis test that determines if the means from two or more samples of data (usually three or more) originate from the same distribution. An F-statistic, also known as an F-test, is a class of statistical tests that use a statistical test like ANOVA to calculate the ratio between variance values, such as the variance from two separate samples or the explained and unexplained variance [38]. An ANOVA F-test is a sort of F-statistic that uses the ANOVA approach, and it can be used to identify the top k most relevant features in a feature selection strategy.

#### Uniform Manifold Approximation and Projection (UMAP)

UMAP (Uniform Manifold Approximation and Projection) is an innovative manifold learning algorithm for dimension reduction, invented by Leland McInnes et al. [39]. Furthermore, the UMAP algorithm arguably conserves the global structure with higher performance and no computational restrictions on embedding dimensions [40]. In addition, UMAP is among the fastest manifold learning applications available, and it consists of two principal stages:

- Creating a graph in high dimensions and calculating the bandwidth of the exponential probability, $\sigma$, through the binary search and the fixed number of the nearest neighbours.
- Applying Stochastic Gradient Descent (SGD) to optimize the low dimensional representation to improve the computation speed. UMAP calculates the exponential probability distribution in high dimensions as:

$$\begin{aligned} p_{i|j} = e^{-\frac{d(x_{i},x_{j}) - p_{i}}{\sigma _{i}}} \end{aligned}$$

where: p represents the distance from each i-th data point to its first nearest neighbour. Moreover, UMAP uses the number of the nearest neighbour’s k as follows:

$$\begin{aligned} p_{ij} = p_{i|j} + p_{j|i} - p_{i|j}p_{j|i} \end{aligned}$$

### Evaluation metrics

To compute the performance of the suggested model, we will evaluate true positives (TP), true negatives (TN), false positives (FP), and false negatives (FN), such as:

- TP: A severe case of COVID-19 is labeled as severe.
- FP: A non-severe case is classified as severe.
- FN: A severe case is classified as non-severe.
- TN: A non-severe is classified as non-severe.

Additional metrics will be computed, including Accuracy, Specificity, Sensitivity, Roc Auc score, and loss. The following formula is used to calculate these figures:

- Accuracy is the ratio of correctly predicted observations. Where:
  $$\begin{aligned} Accuracy = (T P + T N) / All \, predictions \end{aligned}$$
- Specificity is the metric that evaluates a model’s ability to predict true negatives of each available category:
  $$\begin{aligned} Specificity = T N / (T N + F P) \end{aligned}$$
- Sensitivity is the metric that evaluates a model’s ability to predict the true positives of each available category. Where:
  $$\begin{aligned} Sensitivity = T P / (T P + F N) \end{aligned}$$
- Zero-one loss: Standard losses function in classification.
- AUC score: Regardless of the classification threshold chosen, the AUC assesses the quality of the model’s precision and the ranking quality of predictions.

## Experimental results and discussion

### Data processing

The datasets were processed by removing missing values and encoding categorical attributes before machine learning models were deployed. Indeed, we notice that many of the patient entries in the dataset contain missing values. Missing data can be attributed to a variety of factors, including data entry errors, the inability of some patients to attend the clinic, and so on. Data analysis outputs may be wrong and erroneous if missing values are not handled, resulting in bias in later phases and inadequate models used in decision-making processes. Replacing missing values with estimated abundances is considered inappropriate because it introduces knowingly false measurements, such as the presence or absence of elements such as D-dimers, C-reactive protein, and other characteristics of the collected data, which can differ greatly from patient to patient. As a result, the alternative is to delete the samples with missing values, which account for only 3.6% of the data, resulting in a new dataset with 322 entries (Table 2).

**Table 2:** Percent of missing values

Feature with missing values	Percent of missing values
D_dimer	3.6
C_reactive protein	3.6
Weight	1.2

**Fig. 2:**
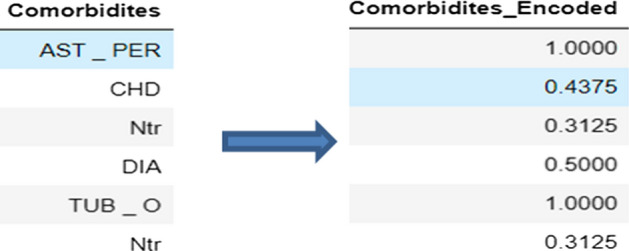
Comorbidities feature before and after encoding

On the other side, we dealt with the dataset’s categorical feature, Comorbidities. To substitute the values of the variable, we apply the Mean Encoding technique while keeping the goal column Y, which is the severity classification, in mind. Because our Dataset’s target Y (Severity Classification) is binary, $Y \epsilon \left\{ 0,1 \right\}$ Mean Encoding algorithm converts all values of the Comorbidities variable into a probability normalized between 0 and 1 (Fig. 2).

### Data visualization and multivariate analysis

**Fig. 3:**
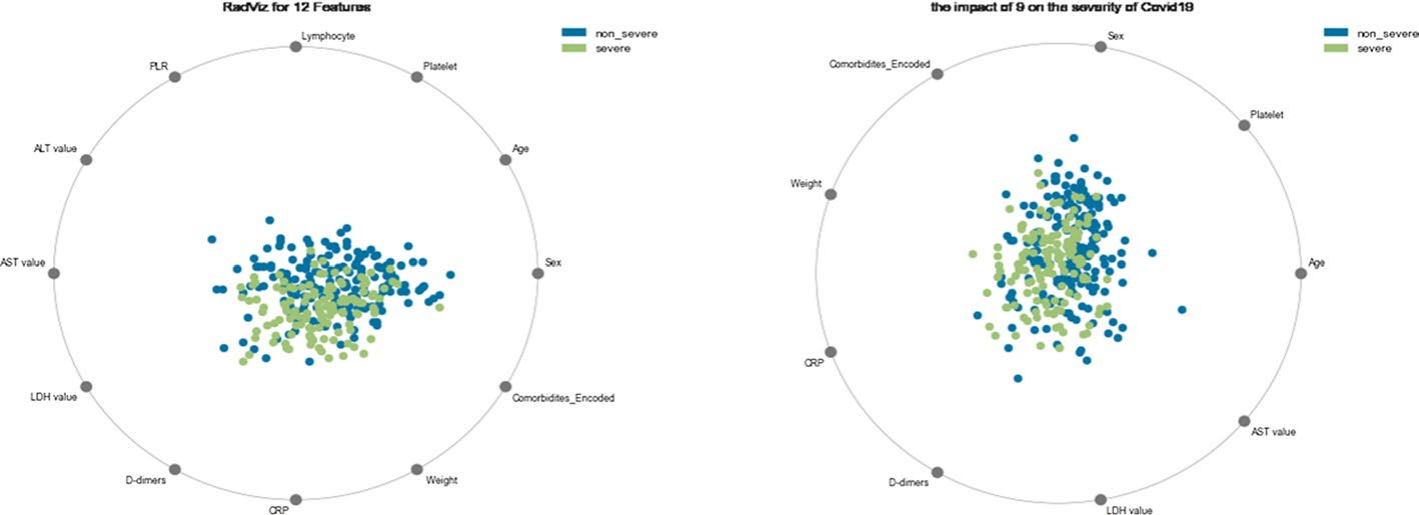
RadViz Visualizer’s main distinguishing features, classified by severity

According the gathered dataset, 54 % of men (43.35 %) had the severe form, and 46% of women (37.58%) had the severe form .According to DeGrace et al. and Ya’qoub et al. [41, 42] Men are the most damaged by COVID-19, because they are more inclined to neglect public health initiatives to control COVID-19. Biological factors such as hormonal, immunological, and inflammatory responses to infection, on the other hand, are responsible for the differences in Covid19 severity between men and women, according to Bulubas [43]. Female hormones (Estrogens) have been demonstrated to boost both innate and adaptive immune responses in women, potentially leading to faster pathogen clearance and less symptoms [44]. Moreover, we can see using the RadViz Visualizer that the COVID-19 severity discriminators in our data included Platelet, Age, Sex, comorbidities, weight, CRP, D dimer, LDH value, and AST value (Fig. 3). However, numerous studies [45–51] have established that the most important markers are CRP, D-dimer, and platelets. That is why, in this study, we will focus on these parameters in order to determine their impact on the severity of Covid 19.

The detection of certain biomarkers associated with viral infection is a more interesting avenue, which can be achieved by simply re-adapting our existing infrastructure; SARS-CoV-2 viral infection triggers various inflammatory, biochemical and haematological biomarkers. Due to the route of infection it takes, the virus causes a significant inflammatory reaction. Accordingly, various inflammatory markers have been reported to be closely associated with this infection, such as C-reactive proteins, interleukin-6, procalcitonin and ferritin. Detection of these biomarkers can simultaneously help understand the disease level of the affected patient [52].

C-reactive protein is a major biomarker present in the bloodstream at the time of infection or inflammation and is produced by liver cells in response to inflammation [53]. CRP levels below 0.3 mg/dL are considered normal in healthy adults [54]. The concentration in COVID-19 patients is said to be higher. A work by Ali et al. [55]. shows that elevated CRP levels may be early indicators of the course of the COVID-19 disease, which is consistent with the result of our study. In the event of infection or inflammation, the levels of this protein increase by approximately 1000 times [56]. It is involved in cardiovascular disease, diabetes and neurodegenerative diseases [57].

Several studies show that CRP increases with severity in patients with COVID-19 [48, 49, 51–55]. The studies identified markers of inflammation, showing the strongest association with the patient’s need for mechanical ventilation and severity which is followed by the CRP peak [59].

Wang et al.report that the initial and peak concentrations of D-Dimer and CRP in the critical group were higher than those in the severe group, the initial and trough counts of lymphocytes were lower than those in the severe group [47].

For Taj et al., the median (IQR) CRP (p-value 0.0001) was higher in patients with severe disease. Platelet count did not show a statistically significant association with disease severity [60]; however, in our study the severity was more important when the platelet count decreased.

**Fig. 4:**
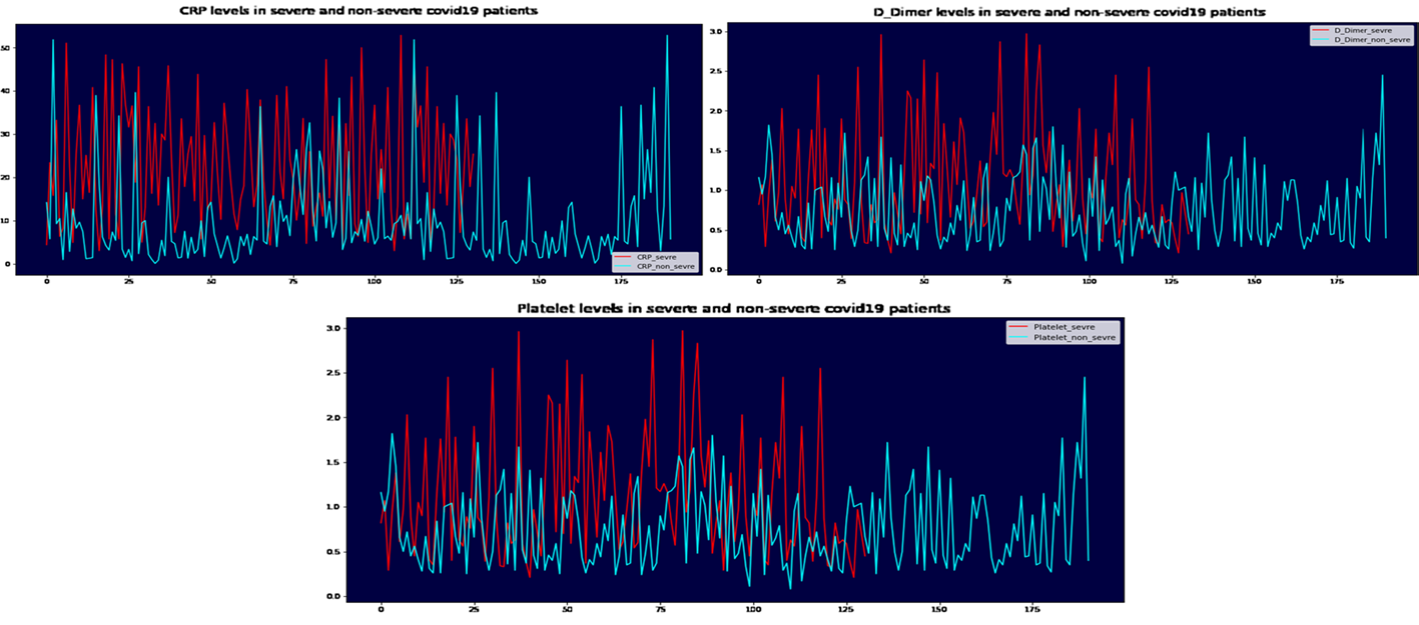
CRP, D-Dimer and Platelet distribution in severe and no severe COVID-19 patients

**Fig. 5:**
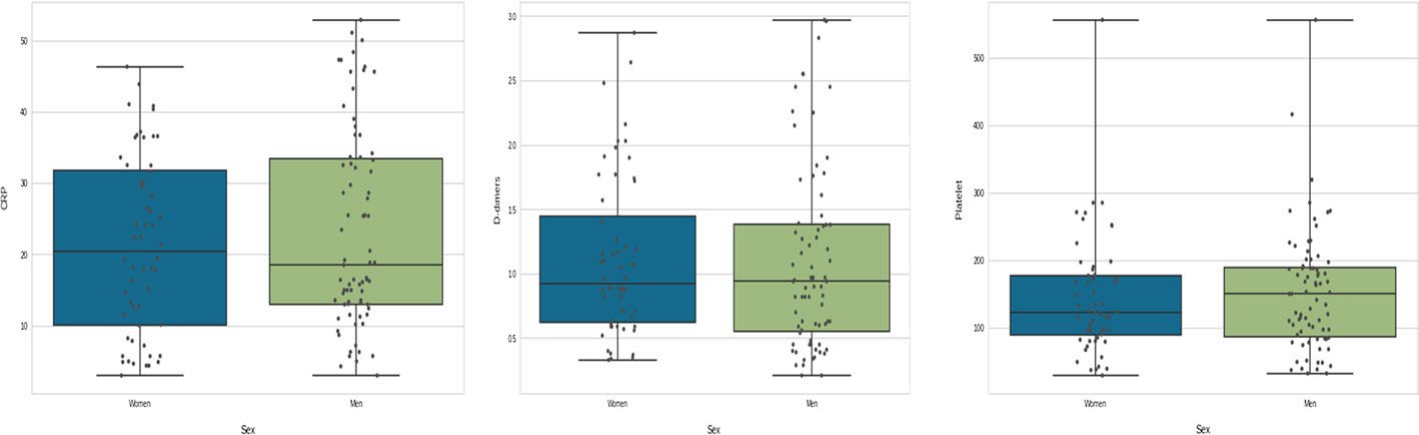
CRP, D-dimer and Platelet rates in women and men suffering from severe COVID-19

According to our findings, CRP levels were higher in severe cases (Fig. 4), particularly in men. The activation of gender-specific T cells is connected to the increased pro-inflammatory response, which increases the probability of COVID-19 infection in male population [61] (Fig. 5).

**Fig. 6:**
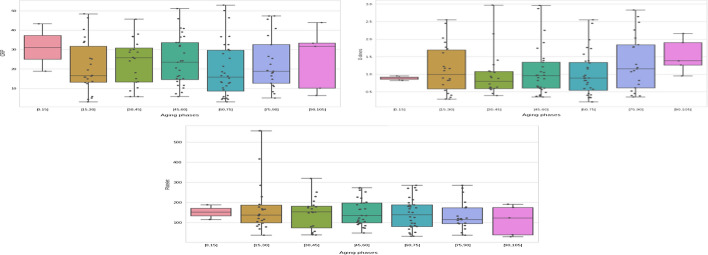
CRP, D-Dimer and Platelet levels in patients with severe COVID-19 at different age groups

COVID-19 severity and CRP readings rose in older males over 60 years old in our group, as in earlier studies (Fig. 6). Reduced testosterone levels in elderly men lead to higher levels of pro-inflammatory cytokines, which may hasten the onset and severity of COVID-19 in these men [62, 63]. CRP is one of these cytokines, and an early elevation in CRP has been used as a predictor of illness severity [64, 65].

On the other hand, D-Dimer is one of the most important aspects in determining severity, which is understandable because patients with severe disease are more likely to have dysregulated coagulation function and a much higher D-dimer level (Figs. 4, 5).

According to Yu et al. [61], patients with severe COVID-19 had a higher level of D-dimer than those with non-severe disease, and D-dimer greater than 0.5 g/ml is related with severe infection in both men and women with COVID-19 (Figs. 4, 5, 6). On the other hand, Ooi et al. [62] discovered that D-dimer readings might be used to guide anticoagulant therapy and prognosis.

Both men and women with severe COVID-19 had reduced platelet counts, according to our data (Fig. 5). Our findings are consistent with those of Seyit et al. [63]. Low platelet counts have also been linked to COVID-19 severity, as per the researches [66–68], and Platelets in COVID-19 patients are higher in men than in women; this difference could be explained by a variety of factors, including biological differences (chromosomal, hormonal, etc.) and gender-specific behavioural factors, as well as pre-existing rates of comorbidities [69, 70].

### Feature engineering

The proposed feature engineering techniques were built and applied throughout the full dataset using Scikit-learn, a freeware Machine Learning toolbox for the Python programming language.To assess these approaches, we utilized a variety of Machine Learning classifiers, including Logistic Regression, Decision Tree, Gaussian NB, SVM, and KNN, which were trained on 70% of the reduced data created by each feature engineering methodology and tested on 30% of that data.

The p-value and Chi Score are two essential outputs of the Chi-square test. When the p-value is larger, it indicates that the input feature is independent of the target and cannot be used in model training.

On the other hand, higher the Chi-Square Score, the feature is more dependent on the response and it can be selected for model training.

**Fig. 7:**
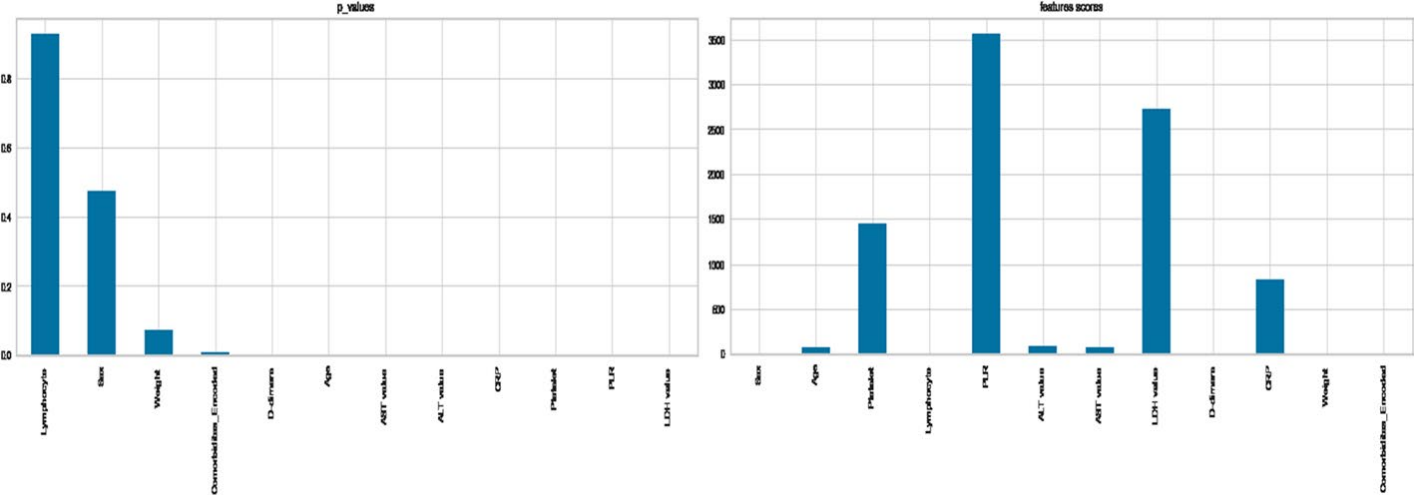
p values for Chi2 tests and Chi2 scores

Figure 7 shows that the greatest p-values are for Sex, Lymphocyte, and Weight, indicating that these factors and the outcome variable are independent. So, as a result, we can get rid of it.

In addition, Platelet, PLR, LDH value, and CRP, have particularly high Chi2 Scores, indicating that the relationship between these variables and the target variable is statistically significant (Fig. 7).

**Fig. 8:**
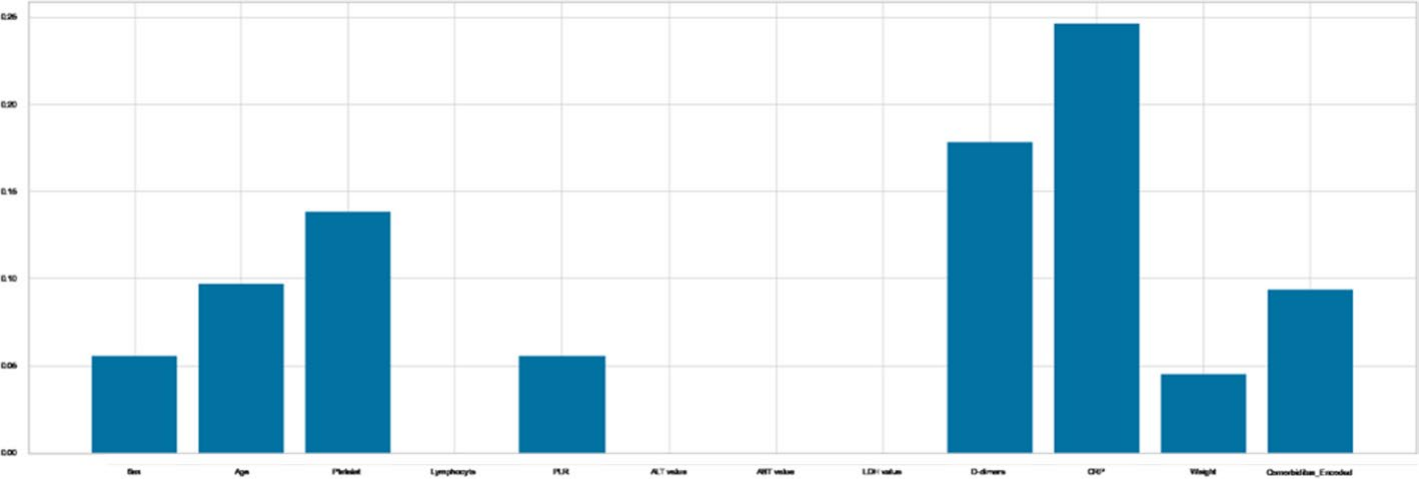
Mutual information scores

On the other hand, as shown in Fig. 8, the Mutual Information scores for the features Platelet, D-dimers, CRP, and Comorbidities are the highest. Others do not, implying that they had no bearing on the classification decision.

**Fig. 9:**
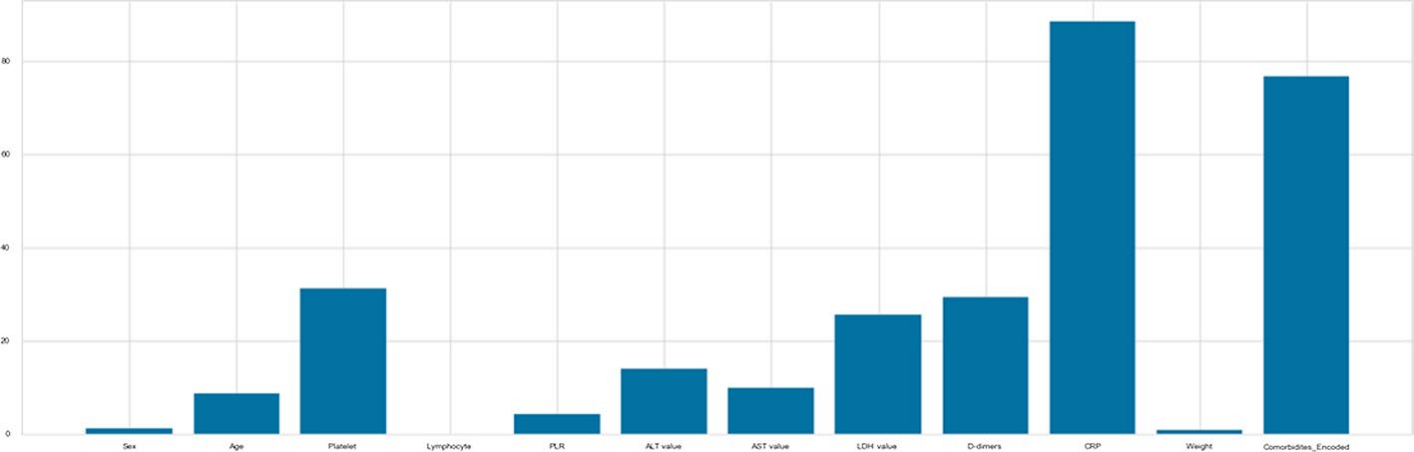
Anova scores

For Anova test, the variance of a feature determines how much it is impacting the response variable. If the variance of a feature is low, it implies there is no impact of this feature on response and vice-versa, and as shown in Fig 9, the attributes Platelet, LDH value, D-dimers, CRP, and Comorbidities, are the features with the highest ratings for Anova,. Others do not, meaning that they had no bearing on the outcome.

**Fig. 10:**
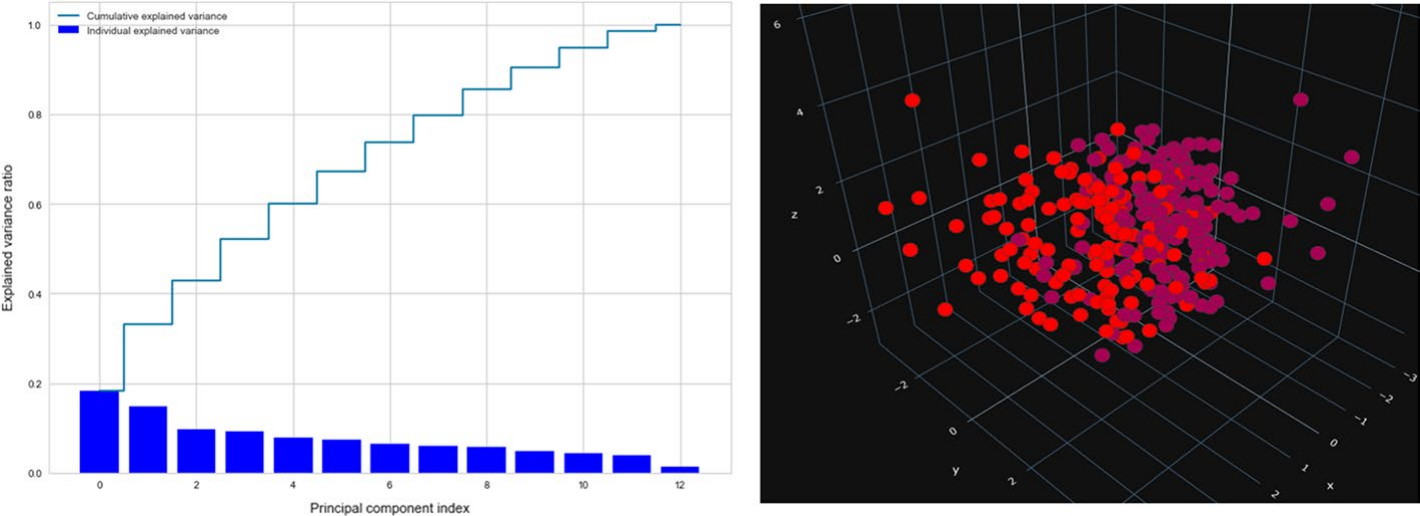
Variance of PCA components and PCA Projection

Moreover,we used the PCA method to calculate the percentage of explained variances of the features, and as shown in Fig. 10, the first three components only reflect 42% of the data, and we can gain 90% of the information with only ten components, negating the need for the PCA approach for reduction.

**Fig. 11:**
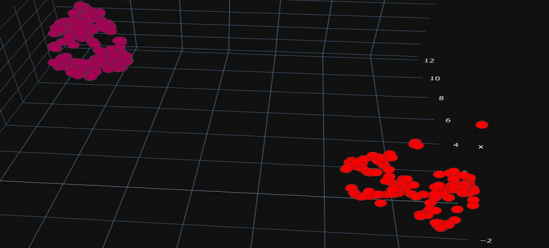
UMAP projection

Finally, Fig. 11 displays the COVID-19 dataset projected in three dimensions using UMAP. As can be seen, UMAP divides the output groups clearly, keeping as much of the local and global data structure as feasible while reducing runtime.

To compare the proposed features engineering approaches, several Machine Learning algorithms are employed, including Logistic Regression, Decision Tree, GaussianNB, SVM, and KNN. For this, we create five subsets, each of which comprises the most significant features according to each feature engineering method (Table 3). Each subset is divided into two groups, one for training and the other for testing (Table 4).

**Table 3:** The various ensembles created through feature engineering methods

Set	Features Selected
Chi square set	Platelet, PLR, LDH value, CRP
Mutual Information set	Platelet, D-dimers, CRP, Comorbidities
Anova set	Platelet, LDH value, D-dimers, CRP, Comorbidities
PCA set	Three components
UMAP set	Three components

**Table 4:** split of each subset in train and test

	Non_severe	Severe
Train set	131	94
Test set	60	37

The performances of Machine Learning algorithms that were applied to each ensemble are shown in Tables 5, 6, 7, 8 and 9.

**Table 5:** Performances of machine learning algorithms applied to Chi square set

Classifier	Accuracy	Sensitivity	Specifity	Loss	AUC
Logistic Regression	0.70	0.76	0.59	0.29	0.74
Decision Tree	0.67	0.66	0.67	0.32	0.67
GaussianNB	0.63	0.6	0.70	0.36	0.73
SVM	0.70	0.75	0.62	0.29	0.73
KNN	0.65	0.7	0.59	0.34	0.71

**Table 6:** Performances of machine learning algorithms applied to mutual information set

Classifier	Accuracy	Sensitivity	Specifity	Loss	AUC
Logistic Regression	0.74	0.78	0.67	0.25	0.81
Decision Tree	0.70	0.81	0.51	0.29	0.67
GaussianNB	0.73	0.8	0.62	0.26	0.79
SVM	0.74	0.81	0.62	0.25	0.79
KNN	0.73	0.78	0.64	0.26	0.8

**Table 7:** Performances of machine learning algorithms applied to Anova set

Classifier	Accuracy	Sensitivity	Specifity	Loss	AUC
Logistic Regression	0.71	0.71	0.70	0.28	0.81
Decision Tree	0.63	0.66	0.59	0.36	0.63
GaussianNB	0.71	0.73	0.67	0.28	0.78
SVM	0.75	0.78	0.70	0.24	0.79
KNN	0.75	0.81	0.64	0.24	0.76

**Table 8:** Performances of machine learning algorithms applied to PCA set

Classifier	Accuracy	Sensitivity	Specifity	Loss	AUC
Logistic Regression	0.71	0.73	0.67	0.28	0.79
Decision Tree	0.65	0.65	0.67	0.34	0.66
GaussianNB	0.70	0.75	0.62	0.29	0.77
SVM	0.71	0.71	0.70	0.28	0.78
KNN	0.64	0.68	0.59	0.35	0.70

**Table 9:** Performances of machine learning algorithms applied to UMAP set

Classifier	Accuracy	Sensitivity	Specifity	Loss	AUC
Logistic Regression	0.98	1.0	0.97	0.01	1.0
Decision Tree	1.0	1.0	1.0	0.0	1.0
GaussianNB	0.98	1.0	0.97	0.01	1.0
SVM	1.0	1.0	1.0	0.0	1.0
KNN	0.98	1.0	0.97	0.01	1.0

**Fig. 12:**
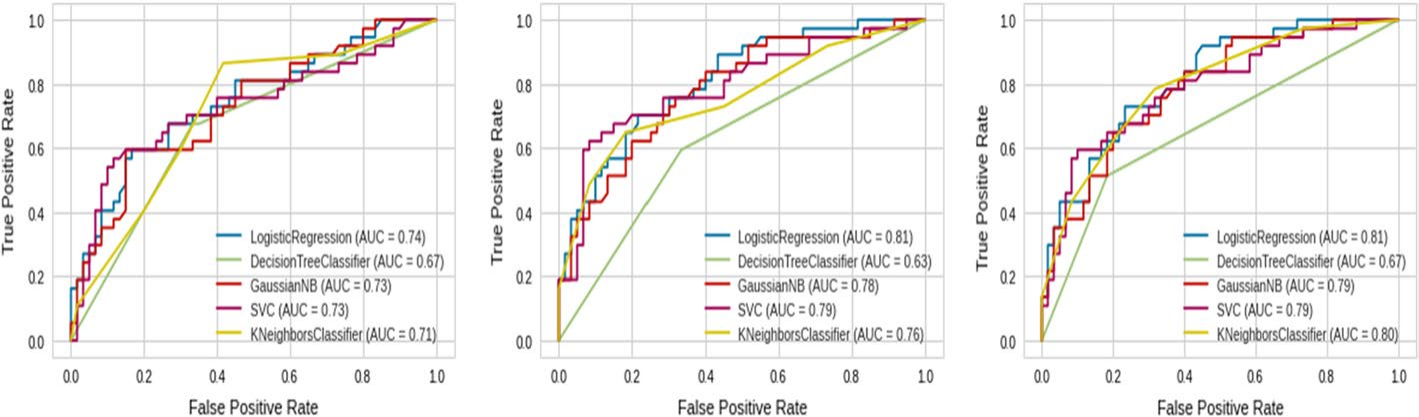
Roc curves of different Machine Learning on the Chi2 set, ANOVA set, and Mutual Information set (from left to right)

**Fig. 13:**
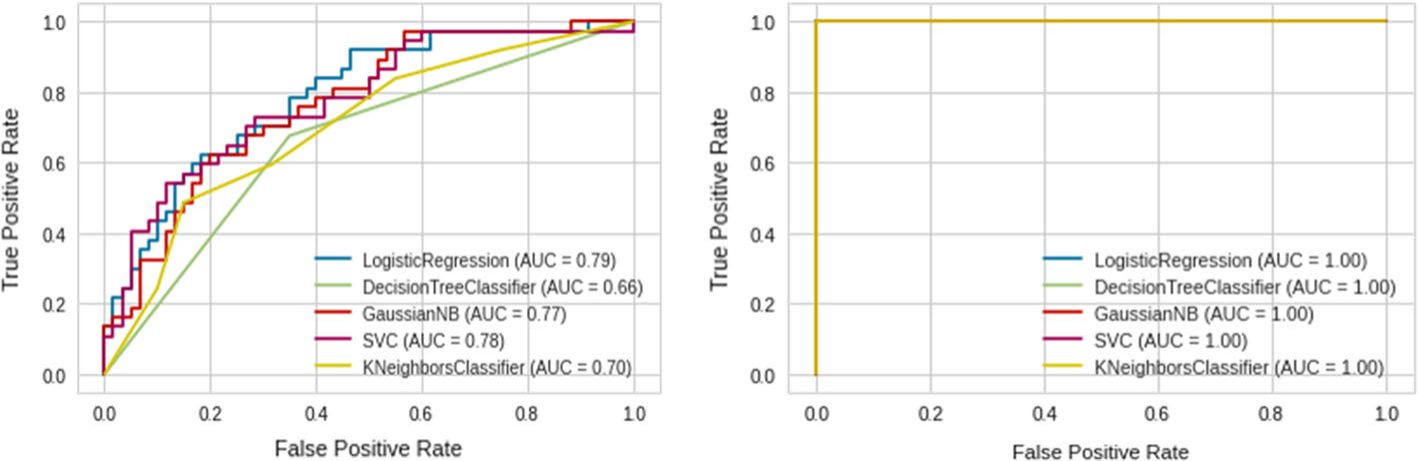
Roc curves of different machine learning on PCA set and UMAP set (from left to right)

### Discussion

When comparing the Chi2 set to the Mutual Information set, the latter is made up of the following characteristics: Platelet, D-dimers, CRP, and Comorbidities, whereas the Chi2 set includes the features: Platelet, PLR, LDH, and CRP, and as shown in Tables 4 and 5 and Fig. 12, the Chi2 findings are worse than Mutual Information, implying that Machine Learning have trouble estimating the severity of COVID-19 based on PLR and LDH properties;and that the Platelet, D-dimers, CRP, and Comorbidities features are more relevant in differentiating the severity of COVID-19.

Moreover, we noted that comorbidities and other signs and symptoms had no significant impact on severity using these techniques. Furthermore, while age was not one of the most predictive factors related to severity in this analysis, it has previously been found to be an essential risk factor in prognosis.

On the other hand, Tables 5, 6, 7, 8, 9 and Figs. 12 and 13 indicate that ML with UMAP produced the best outcomes with only 3 components, which could be due to UMAP’s low noise sensitivity and ability to keep as much of the local and global data structure as possible, making it easier for classifiers to learn by deleting linked features and having smaller dimensions.

As a result, we chose UMAP as a reduction method to extract the embedding features that would be employed throughout the training and testing phases and compare our results to state-of-the-art COVID-19 risk prediction models.

**Fig. 14:**
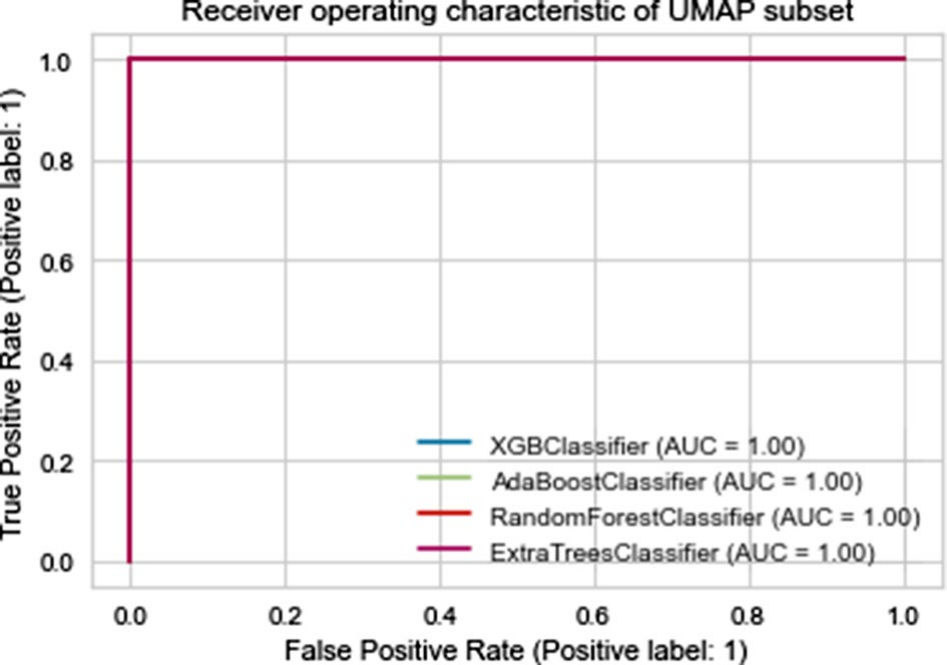
The ROC curve performance of various Machine Learning algorithms in predicting the severity of Covid 19

To accomplish so, we use the XGB Classifier, AdaBoost Classifier, Random Forest, and ExtraTrees models, as well as other machine learning algorithms, to estimate Covid 19 severity. The performance of our model was assessed using various metrics such as accuracy, sensitivity, F-measure, and precision.The results of this experiment phase are shown in Table 10 and Fig. 14.

**Table 10:** The results of our model using additional machine learning

	Accuracy	Sensitivity	Specifity	Loss	AUC
XGB Classifier	1.0	1.0	1.0	0.0	1.0
AdaBoost Classifier	1.0	1.0	1.0	0.0	1.0
Random Forest Classifier	1.0	1.0	1.0	0.0	1.0
ExtraTrees Classifier	1.0	1.0	1.0	0.0	1.0
KNN	0.98	1.0	0.97	0.01	1.0

To assess the analysis of our experimental results, we compared our work to the state-of-the-art COVID-19 risk prediction models listed in Table 11. The main reason for selecting these models is because they produce good findings and use similar datasets, making comparisons more feasible and reliable. Each model’s accuracy, specificity, sensitivity, and AUC values are listed in Table 11. The sensitivity measure piques our interest because it is the most appropriate evaluation in this domain, as misclassifying a severe illness as non-severe results in a substantially larger medical cost than the converse scenario.

**Table 11:** Our model’s performance in comparison to other models

Models	Method of reduction	Model of Prediction	Accuracy	Specifity	Sensivity (%)	AUC
Bayat et al [56]	Features Importance	X_GBoost	86.40%	86.8%	82.39	_
Brinati et al [4]	_	Random Forest	82%	65%	92	84%
Tschoellitsch et al [6]	Feature importance	Random Forest	81%,	82%	60	74%
Tordjman et al [57]	_	Logistic Regression	_	_	80.3	88.9%
Soltan et al [58]	Feature importance	Extreme Gradient Boosted Tree	_	94.8%	77.4	94%
Alakus and Turkoglu [59]	_	LSTM	86.66%	_	99.42	62.50%
Our approach	UMAP	Various Machine Learning	100%	100%	100	100%

## Conclusion

During the severe acute respiratory syndrome-new coronavirus-2 pandemic, clinicians turned to more quick diagnosis approaches due to a lack of laboratory diagnostic instruments and a long wait period. Although techniques based on proteomic analysis can efficiently diagnose COVID-19 at an early stage, it is equally crucial to recognize serious COVID-19 patients before they display severe symptoms.In this study, a set of methods for pre-processing data, manipulating categorical variables, and a feature selection procedure based on various statistical, mathematical and data analysis algorithms was performed to identify the most efficient feature engineering algorithm, for a prognostic prediction of severity. We utilize many Machine Learning algorithms to construct a predictive model to classify the data once pre-processed and reduced.

In terms of accuracy, sensitivity, specificity, and roc curve, the proposed system has proven successful and high performances. Our model may vary if starting from different datasets. As more data become available, the whole procedure can easily be repeated to obtain more accurate models.

This study’s main difficulty is the size of the data. Most of the medical records 337 patients had their information included, but lab results were unavailable for a handful of patients. Another drawback of our model is that it was developed with a predominantly male patient population.

### Future research directions

Additional study in other places will be required to compare the results acquired with other data collected from other laboratories in order to guarantee the accuracy of these results. Furthermore, several decision-making procedures can be utilized to identify patients with distinct degrees of Covid 19 disease severity.

## Data Availability

Not applicable. For any collaboration, please contact the authors.
